# Supplementary material for: Hypothalamic supramammillary neurons that project to the medial septum modulate wakefulness in mice
Source: Commun Biol. 2023 Dec 12;6:1255. doi: 10.1038/s42003-023-05637-w (PMC10716381; doi:10.1038/s42003-023-05637-w)
Supplement: Supplementary file 2 — Supplementary Information [file 42003_2023_5637_MOESM2_ESM.pdf]

Supplementary Information

Hypothalamic supramammillary neurons that project to the medial septum modulate wakefulness in mice

Mengru Liang[1,#], Tingliang Jian[2,3,#], Jie Tao[4,#], Xia Wang[5], Rui Wang[2], Wenjun Jin[2], Qianwei Chen[2], Jiwei Yao[5], Zhikai Zhao[5], Xinyu Yang[5], Jingyu Xiao[6], Zhiqi Yang[2], Xiang Liao[5], Xiaowei Chen[2,7]*, Liecheng Wang[1]* & Han Qin[5,7]*[†]

[1]Department of Anatomy, School of Basic Medical Sciences, Anhui Medical University, Hefei 230032, China

[2]Brain Research Center and State Key Laboratory of Trauma, Burns, and Combined Injury, Third Military Medical University, Chongqing 400038, China

[3]Farm Animal Genetic Resources Exploration and Innovation Key Laboratory of Sichuan Province, Sichuan Agricultural University, Chengdu 611130, China

[4]Advanced Institute for Brain and Intelligence, School of Medicine, Guangxi University, Nanning 530004, China

[5]Center for Neurointelligence, School of Medicine, Chongqing University, Chongqing 400044, China

[6]Department of Anesthesiology, Chongqing University Cancer Hospital, Chongqing 400030, China

[7]Chongqing Institute for Brain and Intelligence, Guangyang Bay Laboratory, Chongqing 400064, China

[#]These authors contributed equally: Mengru Liang, Tingliang Jian, Jie Tao

*Correspondence: xiaowei_chen@tmmu.edu.cn (X.C.), wangliecheng@ahmu.edu.cn (L.W.), qinhan@cqu.edu.cn (H.Q.)

[†]Lead Contact

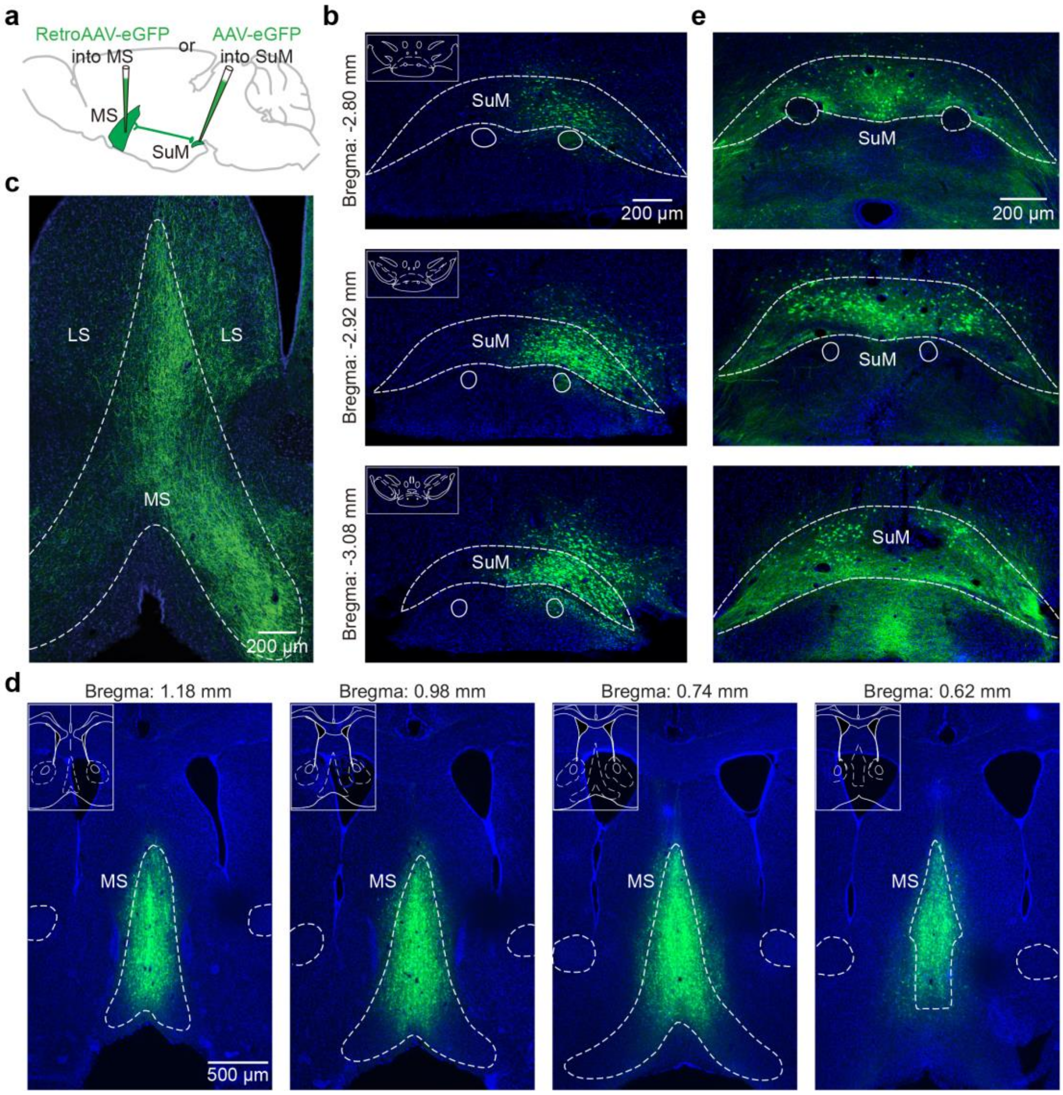

Supplementary Fig. 1 SuM neurons project to MS. a Diagram of AAV-eGFP injection into SuM or retroAAV-eGFP injection into MS. **b** Serial coronal sections illustrating the AAV-eGFP expression at the injection site of SuM. The same experiments were conducted in 5 mice. **c** Representative histological images containing eGFP-labeled axon fibers in MS. **d** Serial coronal sections illustrating the retroAAV-eGFP expression at the injection site of MS. The same experiments were conducted in 6 mice. **e** Representative serial sections showing the eGFP-labeled cell bodies in SuM. SuM, supramammillary nucleus; MS, medial septum; LS, lateral septum.

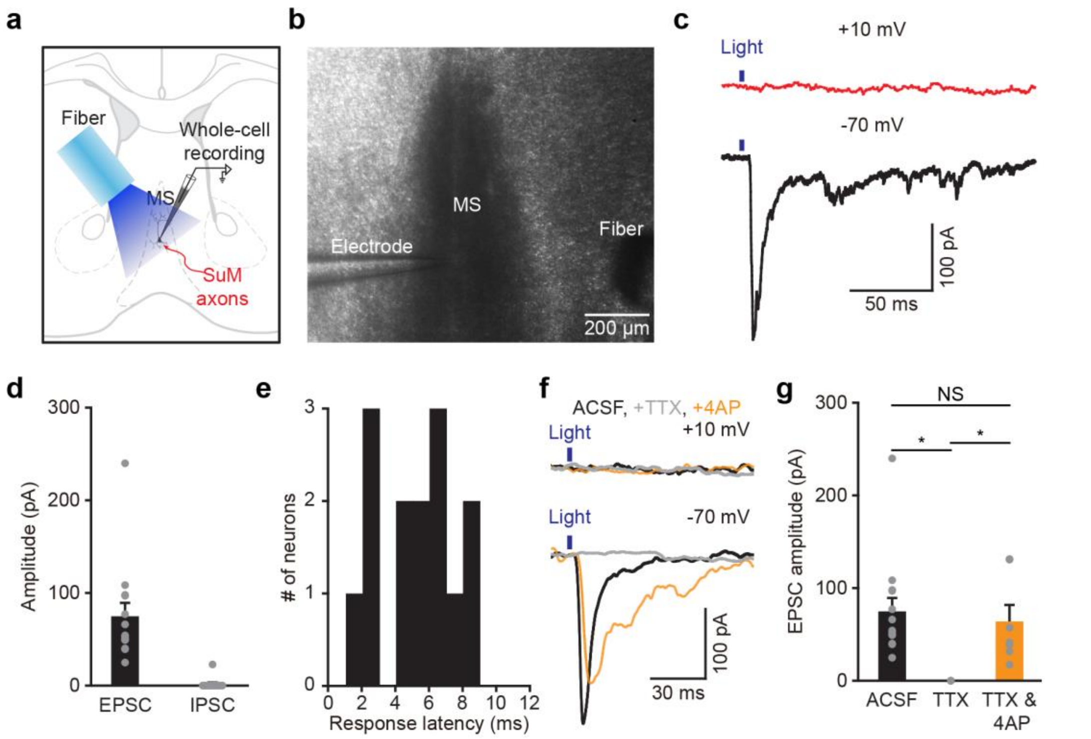

Supplementary Fig. 2 SuM neurons monosynaptically excite MS neurons. a Diagram of whole-cell recordings of MS neurons and SuM axonal fibers undergoing photostimulation in acute brain slices from mice injected with AAV-Syn-ChR2-mCherry. **b** Images showing whole-cell recordings of MS neurons. **c** Sample traces of light-induced inhibitory postsynaptic currents (IPSCs) recorded at +10 mV (red), and excitatory postsynaptic currents (EPSCs) recorded at -70 mV (black). **d** Summary of amplitude for light-induced EPSCs and IPSCs, n = 14 neurons from 4 mice. **e** Distribution of latency for light-induced EPSCs (5.3 ± 0.6 ms), n = 14 neurons from 4 mice. **f** Representative example illustrating the effects of the application of tetrodotoxin (TTX) and 4-aminopyridine (4-AP) on EPSCs recorded from MS neurons. **g** Summary of the application of TTX and 4-AP on light-induced EPSC amplitude from MS neurons; ACSF: data from (**d**), n = 14 neurons, TTX: n = 4 neurons, 4-AP: n = 7 neurons; 1-way ANOVA with LSD post hoc comparison, *$p < 0.05$. Data are represented as mean ± SEM.

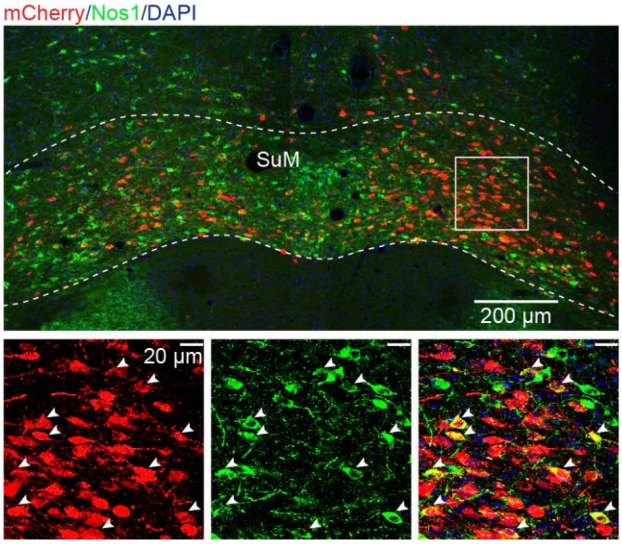

Supplementary Fig. 3 Nitric oxide synthase (Nos1) immunohistochemistry exhibits a subpopulation of SuM^{MS} projecting neurons containing Nos1 (17.6 ± 1.6%, *n* = 8 mice); red, SuM^{MS} projecting neurons, labeled by retroAAV-Cre injected in MS and AAV-DIO-mCherry in SuM; green, anti-Nos1.

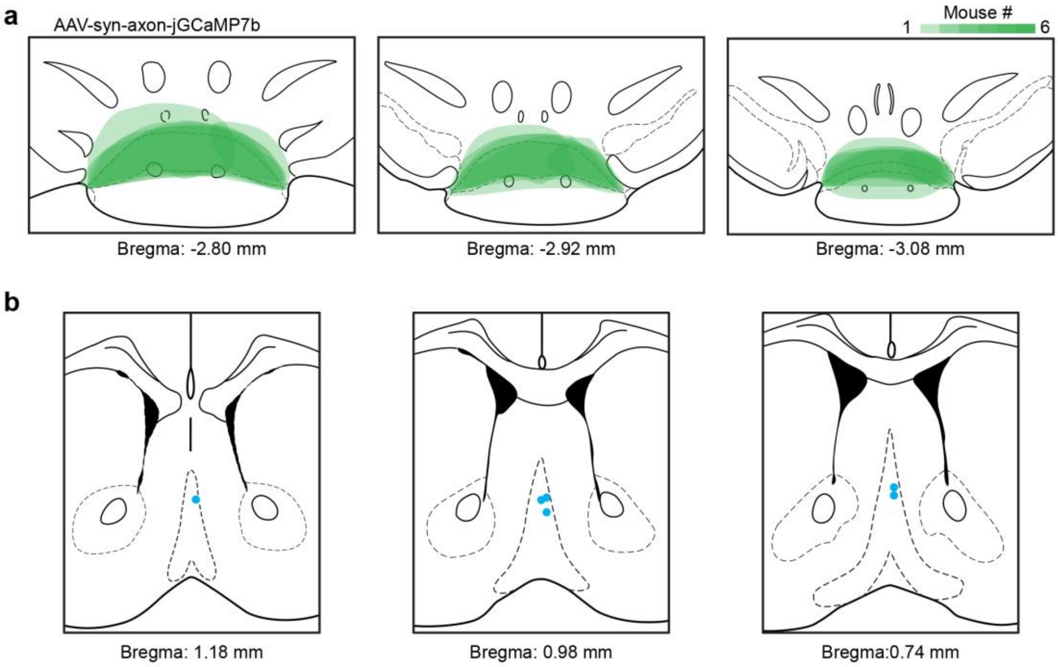

Supplementary Fig. 4 The infection area of AAV-Syn-jGCaMP7b in SuM (**a**), superimposition of all recorded mice, and the fiber tip locations after Ca^{2+} recording in MS (**b**), *n* = 6 mice.

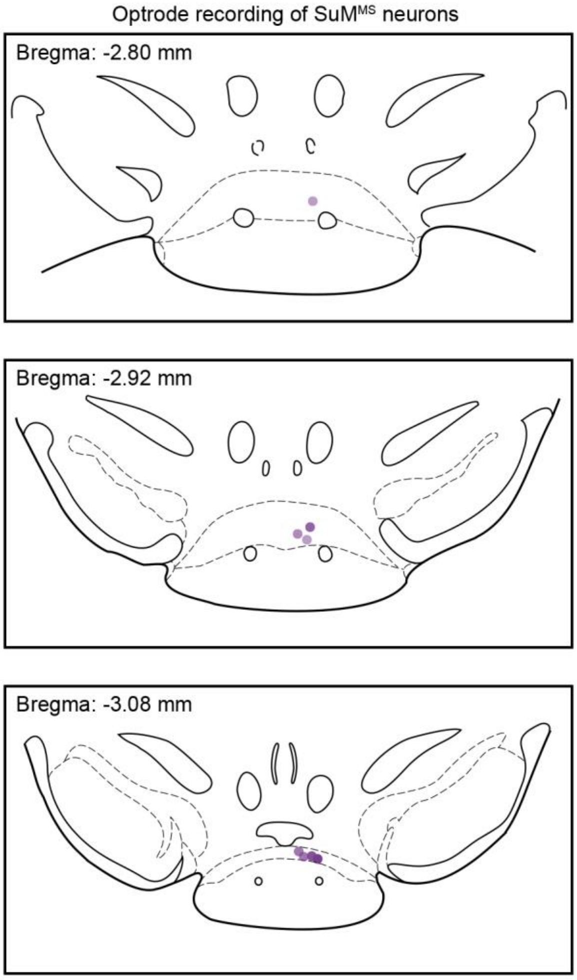

Supplementary Fig. 5 Locations of tetrodes after optrode recordings, *n* = 8 mice.

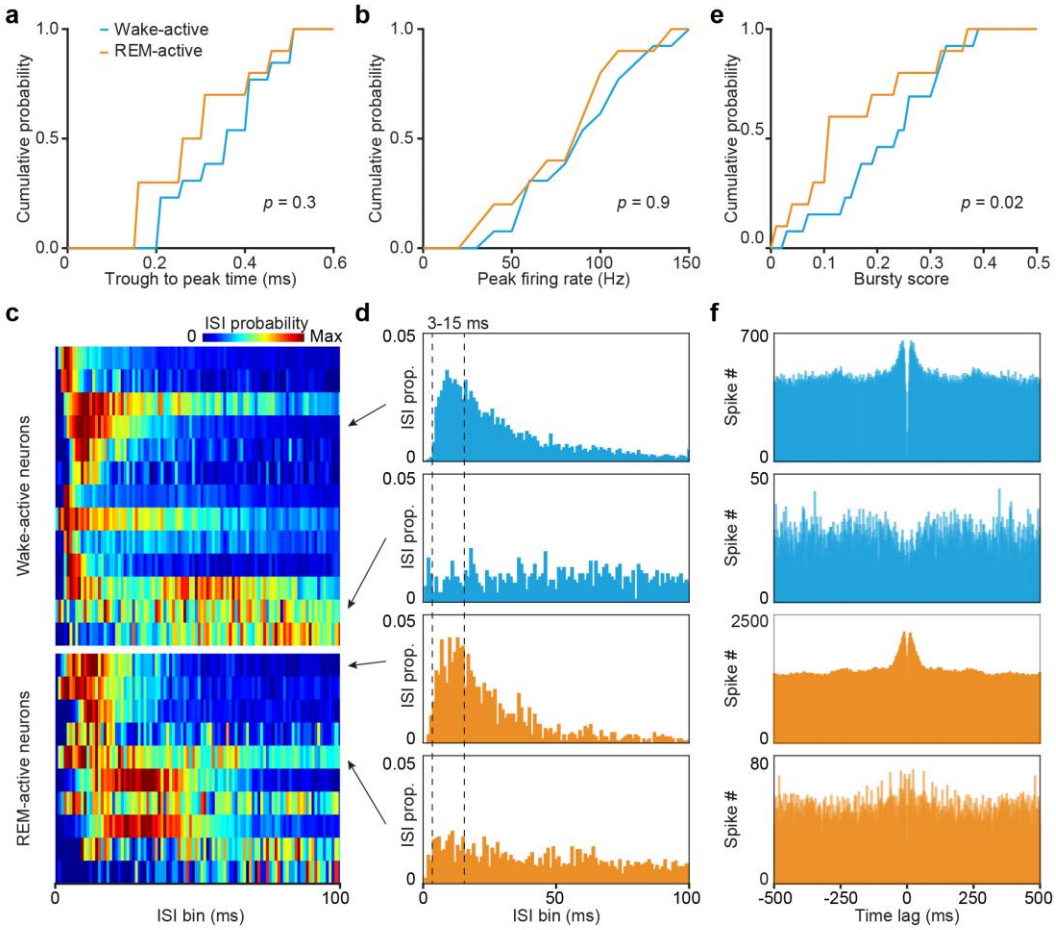

Supplementary Fig. 6 Discharge characteristics of wake-active and REM-active neurons in SuM-MS projection. a-b Cumulative distribution of the trough-to-peak time (**a**, Kolmogorov-Smirnov test, p = 0.3) and peak firing rate (**b**, Kolmogorov-Smirnov test, p = 0.9) for wake-active (blue line, n = 13 neurons) and REM-active neurons (orange line, n = 10 neurons). **c** ISI histograms of all SuM^{MS} projecting neurons, sorted by their respective bursty scores. **d** ISI histograms of two representative wake-active (blue) and REM-active neurons (orange). **e** Cumulative distribution of the bursty score for wake-active (blue line, n = 13 neurons) and REM-active neurons (orange line, n = 10 neurons), Kolmogorov-Smirnov test, p = 0.02. **f** Autocorrelations for the example neurons in (**d**).

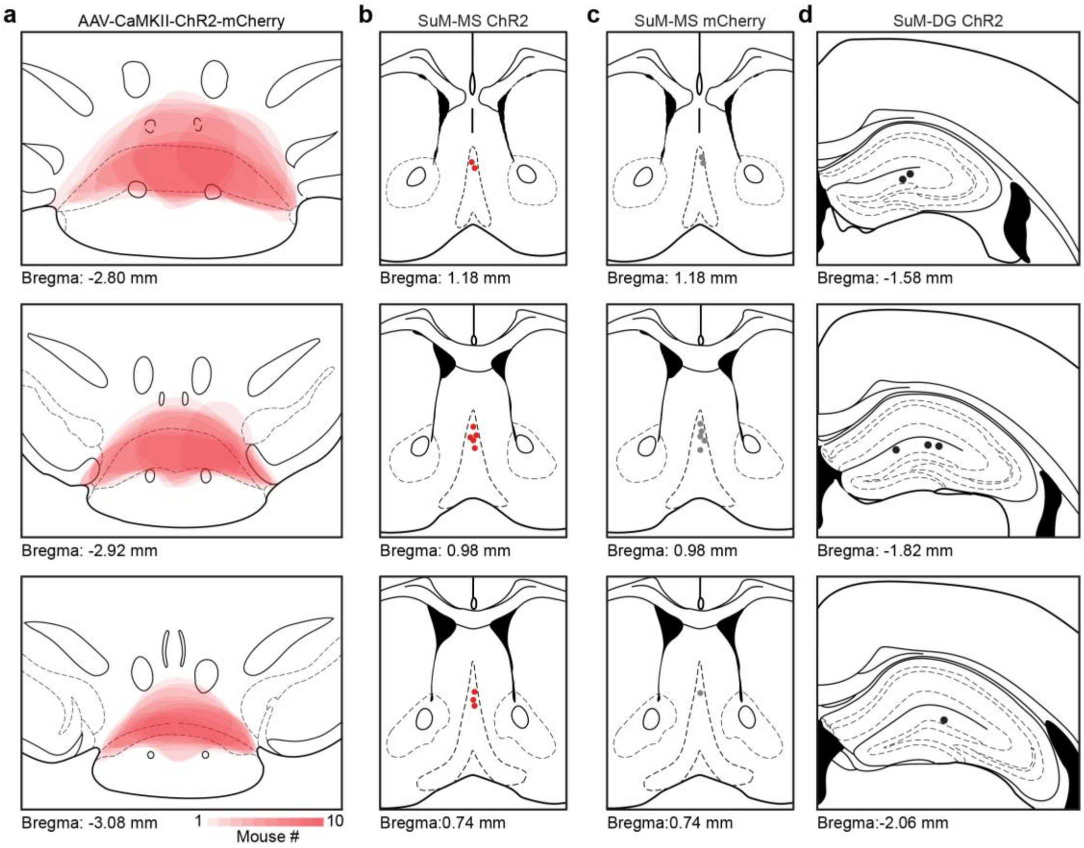

Supplementary Fig. 7 The infection area of AAV-CaMKII-ChR2-mCherry in SuM (**a**, superimposition of 10 mice) and the fiber tip locations after optogenetic activation of the SuM-MS ChR2 group (**b**, *n* = 10 mice), SuM-MS mCherry group (**c**, *n* = 8 mice), and SuM-DG ChR2 group (**d**, *n* = 6 mice).

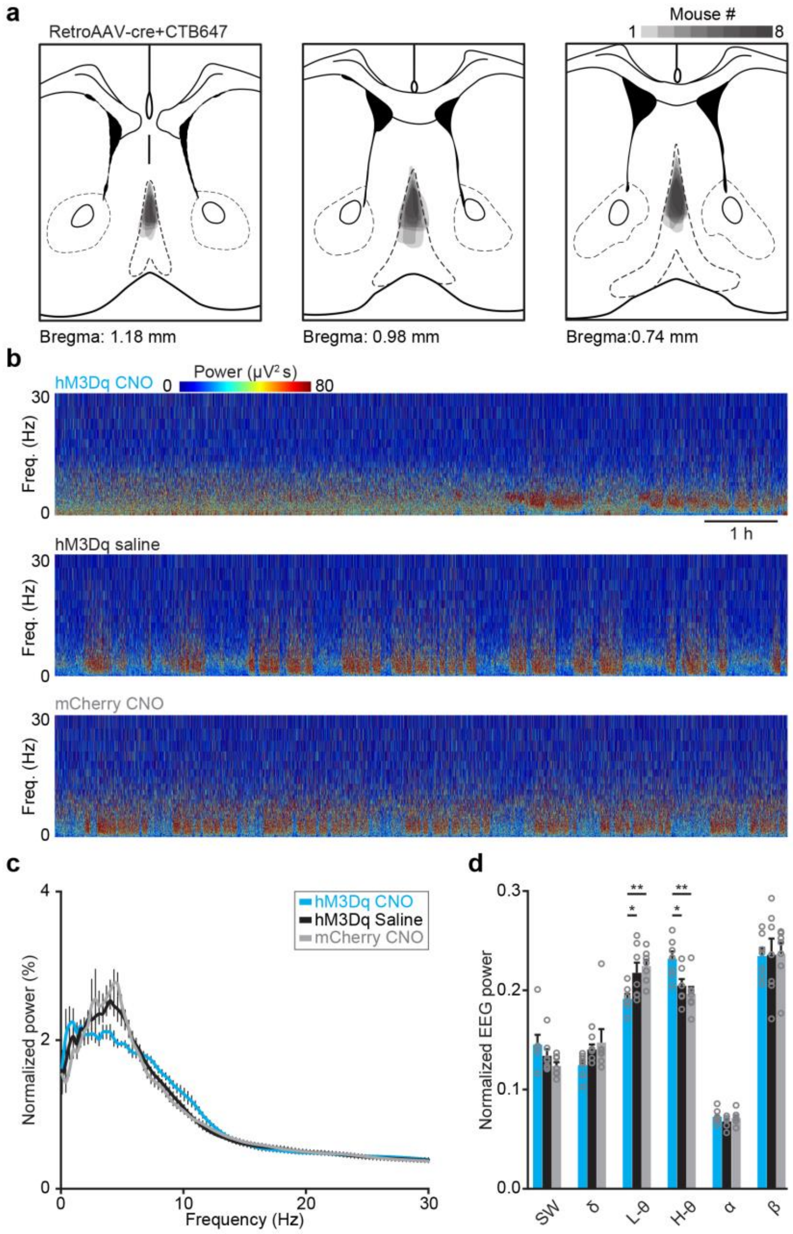

Supplementary Fig. 8 The infection area of retroAAV-Cre in MS and the EEG spectral analysis of chemogenetic activation. **a** Superimposition of the infection range of retroAAV-Cre mixed with cholera toxin subunit B conjugated with Alexa 647 (CTB647), *n* = 8 mice. **b** Representative EEG power spectrum during the 10-hour period after CNO or saline injection in hM3Dq-mCherry or mCherry mice. Freq., frequency. **c-d** Spectral analysis of EEG activity throughout wakefulness after CNO or saline injection of hM3Dq-mCherry or mCherry mice; hM3Dq CNO group, *n* = 7 mice; hM3Dq saline group, *n* = 7 mice; mCherry CNO group, *n* = 7 mice; 1-way ANOVA with LSD post hoc comparison, **p* < 0.05, ***p* < 0.01. Data are represented as mean ± SEM.

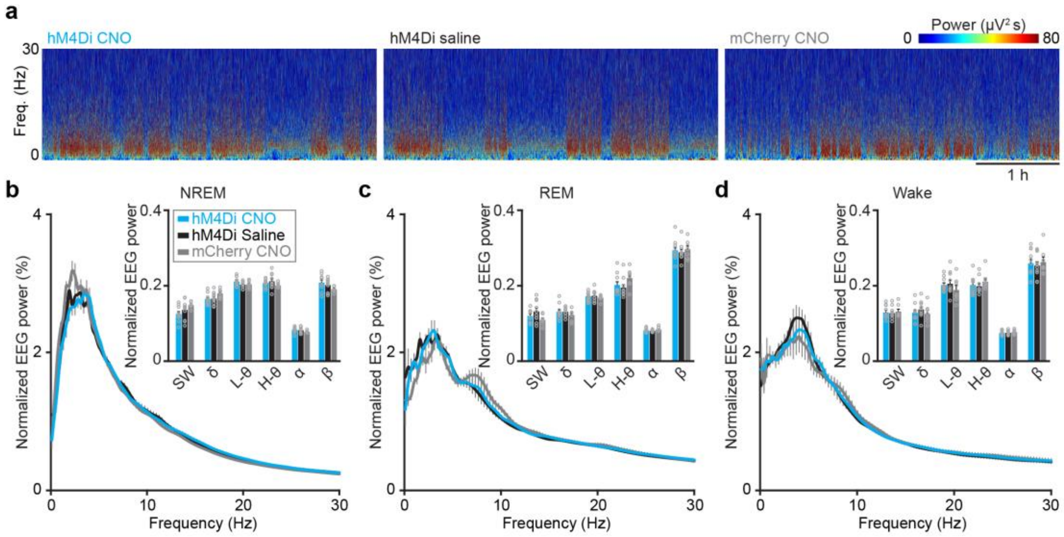

Supplementary Fig. 9 EEG spectral analysis of chemogenetic inhibition. a Representative EEG power spectrum during the 4-hour period after CNO or saline injection in the hM4Di-mCherry or mCherry mice. Freq., frequency. **b-d** Spectral analysis of EEG activity during wakefulness (**b**), NREM (**c**), or REM (**d**) sleep after CNO or saline injection of hM4Di-mCherry or mCherry mice; hM4Di CNO group, *n* = 7 mice; hM4Di saline group, *n* = 7 mice; mCherry CNO group, *n* = 7 mice; 1-way ANOVA test. Data are represented as mean ± SEM.

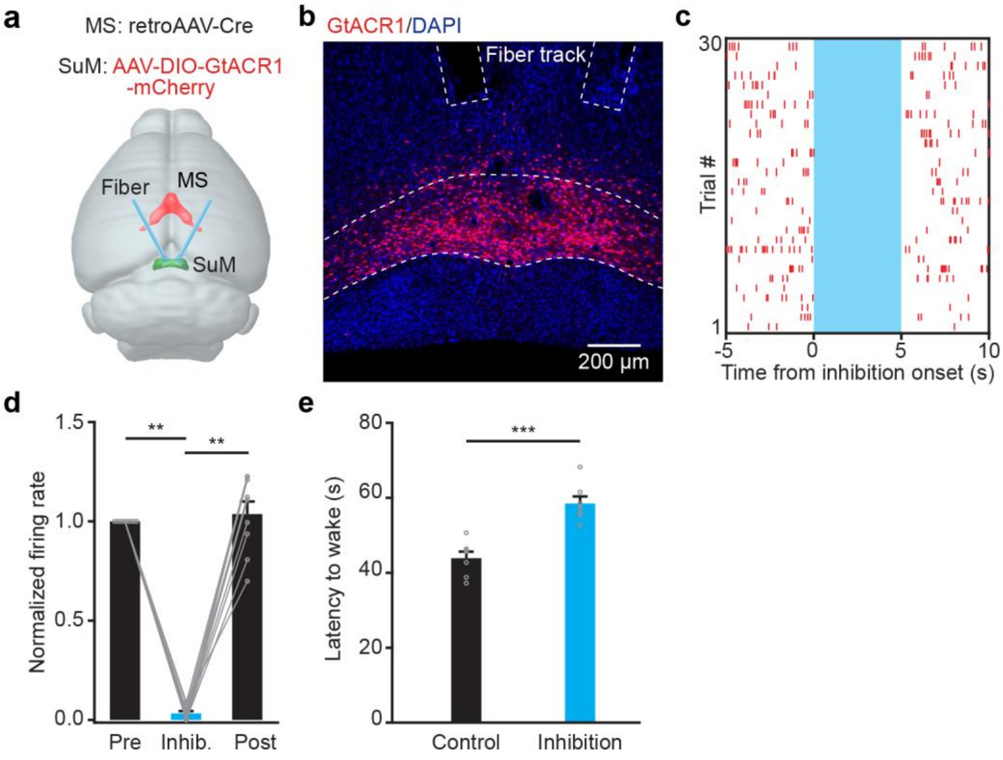

Supplementary Fig. 10 Optogenetic inhibition of SuM^{MS} projecting neurons prolongs sleep. a Experimental design of retrograde labeling of SuM^{MS} projecting neurons with GtACR1, fiber implantation into SuM, and EEG-EMG recording. **b** Sample histological image illustrating the expression of GtACR1 in SuM^{MS} projecting neurons as well as the optical fiber tracks. **c** Raster plot of spikes in an example SuM^{MS} projecting neuron recorded using an optrode exhibiting the silencing effect exerted by light delivery. **d** Summary of light-induced silencing on firing rates of SuM^{MS} projecting neurons recorded by an optrode in freely-behaving mice. Friedman's ANOVA test with post hoc comparison, n = 9 neurons from 3 mice, $^{**}p < 0.01$. **e** Summary of the latency to wakefulness from sleep with optogenetic inhibition. paired *t-test*, n = 7 mice, $^{***}p < 0.001$. Data are represented as mean ± SEM.
