# Supplementary material for: Hypothalamic supramammillary neurons that project to the medial septum modulate wakefulness in mice
Source: Commun Biol. 2023 Dec 12;6:1255. doi: 10.1038/s42003-023-05637-w (PMC10716381; doi:10.1038/s42003-023-05637-w)
Supplement: Supplementary file 3 — Description of Additional Supplementary Files [file 42003_2023_5637_MOESM3_ESM.pdf]

Description of Additional Supplementary Files

File name: Supplementary Data 1
Description: The Statistics Summary in the paper.

File name: Supplementary Data 2
Description: The source data behind the graphs in the paper.
